# Development and Validation of a CT Radiomics-Deep Learning Model for Predicting Surgical Difficulty in Pancreatic and Periampullary Tumors

**DOI:** 10.3390/cancers18010029

**Published:** 2025-12-21

**Authors:** Tao Hu, Yuan Sun, Yan Li, Ming Li

**Affiliations:** Department of Hepatobiliary Surgery, The First Affiliated Hospital of Chongqing Medical University, NO. 1 Youyi Road, Yuanjiagang, Yuzhong District, Chongqing 400016, China; 2023150447@stu.cqmu.edu.cn (T.H.); 2023120494@stu.cqmu.edu.cn (Y.S.); 2023120470@stu.cqmu.edu.cn (Y.L.)

**Keywords:** radiomics, deep learning, computed tomography, surgical difficulty, laparoscopic pancreatoduodenectomy

## Abstract

**Background:** Pancreatic and periampullary cancers are common tumors of the digestive tract. As a radical surgical approach, laparoscopic pancreaticoduodenectomy requires crucial preoperative assessment of its surgical difficulty. **Materials and methods:** A retrospective cohort of 150 patients who underwent LPD between June 2019 and June 2023 was enrolled. The criteria for defining the difficult group were identified as unplanned conversion to open procedure, intraoperative blood loss, and operative time. Participants were randomly allocated to a training set (*n* = 105) or a testing set (*n* = 45) in a 7:3 ratio. Hand-crafted radiomics (HCR) features and deep learning-derived radiomics (DLR) features were extracted from portal venous phase CT images, focusing on gross tumor volume and gross peri-tumor volume. A hybrid prediction model was developed using a support vector machine algorithm, with performance evaluated through receiver operating characteristic analysis, calibration curves, and decision curve analysis (DCA). **Results:** The combined model demonstrated significantly superior discriminative ability, achieving an area under the curve (AUC) of 0.942 (95% CI: 0.893–0.992) in the training set and 0.848 (95% CI: 0.738–0.958) in the testing set. This performance exceeded both the standalone HCR model (testing AUC = 0.754) and the DLR model (testing AUC = 0.816). DCA further confirmed the clinical utility of the combined model, showing the highest net benefit across threshold probabilities exceeding 20%. **Conclusions:** The novel integrated model combining hand-crafted and deep learning-derived radiomics features enables effective prediction of surgical difficulty in laparoscopic pancreaticoduodenectomy.

## Simple Summary

In this retrospective study, we developed and validated an integrated CT radiomics-deep learning model (RDLM) for preoperative prediction of LPD surgical difficulty. The model combines hand-crafted radiomics features (intratumoral and peritumoral) and deep learning-derived features, achieving a test set AUC of 0.848 and high sensitivity (0.850) for identifying difficult cases. Key strengths include non-invasiveness, robust calibration, and clinical net benefit. Contextualized within the field, this model addresses the unmet need for preoperative risk stratification in LPD, complementing existing surgeon-dependent assessments.

## 1. Introduction

Laparoscopic pancreaticoduodenectomy (LPD) has emerged as the primary surgical modality for the treatment of malignant tumors of the pancreatic head, ampulla of Vater, and distal common bile duct [1,2,3]. During this procedure, surgeons often encounter challenges such as tumor infiltration or severe adhesions, which may lead to massive intraoperative bleeding or the need for conversion to open surgery [4]. Operative time, estimated blood loss (EBL), and conversion rate to open surgery are generally recognized as key indicators for evaluating the difficulty of minimally invasive surgery [5,6,7]. In recent years, factors influencing the difficulty of LPD have garnered increasing attention in the field. Studies have demonstrated that factors such as preoperative inflammatory status, previous abdominal surgery history, gender, body mass index (BMI), and tumor growth characteristics all contribute to surgical difficulty [6,8,9,10]. High-difficulty surgeries are often associated with an increased risk of postoperative complications, including pancreatic fistula, bleeding, and surgical site infection (SSI) [6,11,12]. Therefore, accurate preoperative prediction of surgical difficulty holds significant clinical importance for reducing postoperative complications. Nowadays, numerous studies have integrated imaging features with clinical indicators to achieve favorable predictive performance for postoperative pancreatic fistula (POPF) [13,14,15]. These objective structural variables assist surgeons in establishing reasonable preoperative expectations regarding operative duration, bleeding risk, and lymph node dissection scope [16]. In summary, precise preoperative difficulty assessment is crucial for formulating individualized surgical plans and optimizing perioperative management [5,11].

In recent years, computed tomography (CT)-based radiomics has been increasingly applied to the non-invasive preoperative assessment of tumor heterogeneity and surgical difficulty [17,18,19]. Relevant imaging biomarkers include arterial and venous phase enhancement patterns, the latter of which is highly correlated with the degree of peritumoral inflammatory infiltration [20]. Meanwhile, gross peritumoral volume (GPTV) can reflect aggressive biological characteristics such as tumor dissemination patterns and tissue fibrosis [9,21,22]. With the advancement of artificial intelligence (AI) technology, deep learning models can automatically segment raw CT images and extract high-dimensional complex features [23,24], including microstructural features of the tumor-vascular interface and peritumoral fat infiltration. For instance, a study by Wen Liu et al. demonstrated that a hybrid model integrating GPTV_DL, radiomics-based GPTV, and imaging findings can effectively predict lymphovascular invasion status in breast cancer patients preoperatively [25]. In another study, Wenlun Wang et al. confirmed that a decision fusion-based model, which integrates 3D deep learning, 2D deep learning, radiomics, and clinical data, exhibits favorable performance in predicting occult lymph node metastasis in laryngeal squamous cell carcinoma [26]. These studies suggest that a multimodal fusion strategy, which combines manually extracted radiomics features with deep learning features, can enhance model robustness through information complementarity [27,28], thereby serving as a basis for evaluating diagnostic and therapeutic strategies in complex clinical settings. However, the application of multimodal deep learning approaches in the field of LPD surgical difficulty prediction remains in its preliminary stage and requires further in-depth exploration.

Therefore, this study developed a novel integrated model that integrates manually extracted radiomics (HCR) features and deep learning-derived radiomics (DLR) features of gross tumor volume (GTV) and gross peritumoral volume (GPTV), for the preoperative prediction of LPD surgical difficulty, thereby better guiding surgeons in clinical decision-making.

## 2. Materials and Methods

### 2.1. Research Cohort

This retrospective study was approved by the Ethics Review Committee of the First Affiliated Hospital of Chongqing Medical University (Approval No.: 2025-409-01) and strictly adhered to the principles of the Declaration of Helsinki. The requirement for informed consent was waived for all participants.

**Figure 1:**
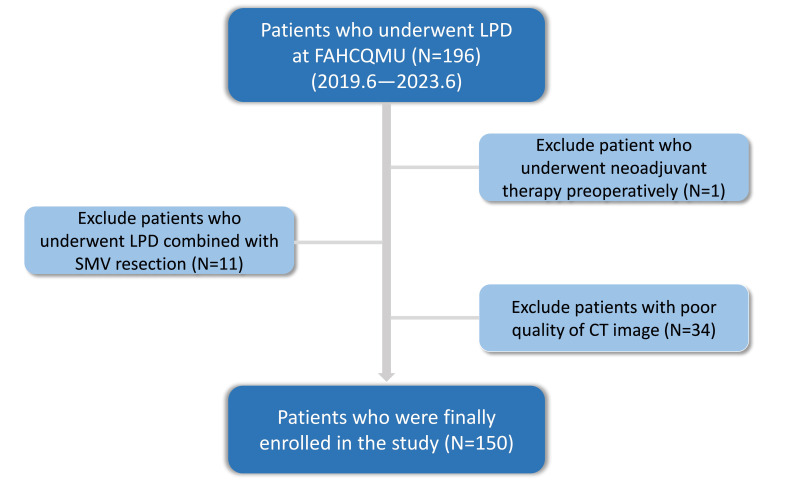
Inclusion and exclusion criteria of the study cohort.

We enrolled 196 patients who underwent laparoscopic pancreatoduodenectomy (LPD) at FAHCQMU between June 2019 and June 2023. Inclusion criteria comprised: (1) availability of complete clinical and pathological data, (2) preoperative contrast-enhanced CT performed ≤1 month before surgery and (3) standard surgical technique [29]. Exclusion criteria were: (1) Superior mesenteric vein (SMV) resection combined, (2) receipt of neoadjuvant therapy, (3) suboptimal CT image quality. After exclusions, 150 patients were included (Figure 1). Based on previous definitions of laparoscopic abdominal surgery difficulty, we introduced the following relevant parameters as the surgical difficulty criteria for laparoscopic pancreaticoduodenectomy (Table 1). Finally, enrolled patients were stratified by surgical difficulty according to a validated classification system [6,7,30,31,32].

### 2.2. CT Technique

Contrast-enhanced abdominal CT scans were performed using Siemens SOMATOM Force(Siemens Healthineers GmbH; Shanghai, China), GE Discovery CT750 HD (GE HealthCare Technologies Inc.; Beijing, China), or GE LightSpeed VCT. Scanning parameters: 120 kV, 200 mA, 5 mm slice thickness. All images were reconstructed using a standard reconstruction kernel with the following parameters: pitch of 1, rotation time of 0.5 s, field of view of 350 mm × 350 mm, matrix size of 512 × 512, slice thickness of 5 mm, interval of 5 mm, and reconstruction slice thickness of 1 mm. Patients were required to fast and avoid drinking for at least 3 h prior to the examination. A nonionic iodinated contrast agent (300–400 mgI/mL) was administered intravenously at a dose of 1–1.5 mL/kg with an injection rate of 3 mL/s. Arterial phase scanning was delayed by 15–18 s. Portal venous and delayed phase scans were performed with delays of 33–36 s and 180 s, respectively. Enhanced CT images were exported from the Picture Archiving and Communication System (PACS) in DICOM format for further analysis.

### 2.3. Image Processing

All images underwent preprocessing, including noise reduction, normalization, and data augmentation. Artifacts from scanning were removed, and samples exhibiting suboptimal image quality or ambiguous labels were excluded. The fixed resolution resampling method was used in our experiment to handle the aforementioned problems. All images were resampled to a voxel size of 1 × 1 × 1 mm to standardize the voxel spacing. Finally, images were standardized by normalizing grayscale values to a 0–255 range. Contrast enhancement, sharpening, and denoising algorithms were subsequently applied to accentuate tumor-region details. Two experienced radiologists manually delineated regions of interest (ROIs), encompassing tumor boundaries and adjacent high-risk areas (e.g., potential infiltration/inflammatory zones), using ITK-SNAP (v3.6.0; https://www.itksnap.org, accessed on 24 April 2024). Annotated images were saved as NIFTI-format masks. GPTV was achieved by radially dilating the original ROI and extending it by 3 voxels beyond the tumor boundaries. To validate ROI reliability and consistency, a senior abdominal radiologist (10 years’ experience) verified segmentations through random inspection of 50 samples. Intraclass correlation coefficients (ICC) quantified feature repeatability and stability, with values >0.8 indicating satisfactory consistency.

#### 2.3.1. Hand-Crafted Radiomics Feature Extraction

Radiomics features were extracted from volumes of interest (VOIs) using PyRadiomics (v 3.0.1; https://pyradiomics.readthedocs.io, accessed on 28 May 2024) [33]. A total of 1834 features were extracted and categorized as intratumoral or peri-tumoral, comprising 360 first-order features, 14 shape-based features, and 1460 texture features. These features characterize morphological, textural, and signal-intensity properties, specifically: first-order statistics, shape-based metrics, and texture parameters from Gray-Level Co-occurrence Matrix (GLCM), Gray-Level Run Length Matrix (GLRLM), Gray-Level Dependence Matrix (GLDM), Gray-Level Size Zone Matrix (GLSZM), and Neighborhood Gray-Tone Difference Matrix (NGTDM). To enhance feature robustness, voxel intensities underwent: (1) six nonlinear transformations (Square, SquareRoot, Logarithm, Gradient, Exponential, and 3D Local Binary Patterns [LBP3D]); (2) Laplacian of Gaussian (LoG) filtering (σ = 1.0, 2.0, 3.0); and (3) wavelet decomposition using eight patterns (LLL, LLH, LHL, LHH, HLL, HLH, HHL, HHH) applied to first-order and texture features [34].

#### 2.3.2. Deep Learning Feature Extraction

All abdominal CT images underwent automated segmentation via deep learning.

Architecture: The 2D convolutional neural network (2D CNN) was fine-tuned on the ImageNet dataset (https://image-net.org/, accessed on 18 September 2024). The slice with the maximum cross-sectional area of the tissue was cropped as the model input, while retaining the batch normalization design of the pre-trained ResNet18. Input images were normalized following the ImageNet protocol to align with the distribution of pre-trained data. This ResNet18 architecture consists of one initial convolutional layer, one subsequent max-pooling layer, four residual blocks, as well as one global average pooling layer and one fully connected layer at the network terminus.

Data augmentation: Random augmentation was applied during training to enhance the generalization ability of the network, increase dataset diversity, and mitigate the risk of overfitting. To preserve the consistency of spatial relationships, random horizontal flipping and random axial rotation with a maximum range of [−5°, +5°] were implemented. For each patient in the training set (across all time points), random augmentation was performed to augment the dataset fourfold. The probability of random horizontal flipping was set to 0.5, and that of random axial rotation was set to 0.4.

Training-validation partitioning: 10-fold stratified cross-validation was adopted to ensure balanced class distribution. The dataset was randomly split into 10 non-overlapping subsets, with each fold containing 80–90 training samples and 35–45 validation samples.

Hyperparameters: The Stochastic Gradient Descent (SGD) optimizer was selected. The initial learning rate was optimized to 0.001 (from candidate values of 0.01, 0.001, and 0.0001) to balance convergence speed and stability. It was then linearly decayed from 0.001 to 0 over 50 training epochs to accommodate convergence requirements in the late training phase. The batch size was set to 96 to balance batch stability and training speed. The total number of training epochs was fixed at 50, and all hyperparameters were kept consistent across the 10 folds to ensure fairness of comparison.

During the model training phase, the 2D CNN was initialized with pre-trained weights for feature extraction and training. The average pooling layer was selected to extract deep features, yielding 512-dimensional deep features and completing the screening of deep learning features.

### 2.4. Feature Selection

Prior to feature selection, all features were normalized using the z-score method. Both types of features were filtered using four steps. First, the Mann–Whitney U test was conducted for all features, wherein only features with a *p*-value < 0.05 were kept. Second, the Pearson test was used to evaluate the correlation between features and categories, and features with a *p*-value < 0.05 were considered potentially predictive. Finally, the key features were screened using the least absolute shrinkage and selection operators (LASSO) [25,35]. The LassoCV framework automatically tuned the regularization parameter via 10-fold cross-validation, optimizing model generalizability while ensuring feature stability.

### 2.5. Predictive Model Development

Support Vector Machines (SVMs) improve model generalizability through margin maximization, exhibiting high noise tolerance and robustness [36]. SVM model was constructed (class_weight = ‘balanced’, probability = True, random_state = 0).The feature sets retained from four distinct filtering methods—traditional radiomics (T: 11 features), peri-tumoral radiomics (P: 10 features), deep learning radiomics (DL: 19 features), and their combination (Combined: 15 features)—were utilized as the final input variables for the SVM model. Class imbalance was mitigated using the Synthetic Minority Over-sampling Technique (SMOTE). To mitigate overfitting, three strategies were employed during model optimization: maximum iteration limits, L2 regularization, and early stopping. The training set was partitioned into an 80% training subset and a 20% test subset via 5 random splits, with roc_auc_score employed as the evaluation metric to identify the optimal data partition and its corresponding model. The final predictive model was selected through five-fold cross-validation.

### 2.6. Performance Evaluation

The predictive performance of radiomics and deep learning models was evaluated using multiple metrics: area under the curve (AUC), diagnostic accuracy, sensitivity, specificity, positive predictive value (PPV), and negative predictive value (NPV). Receiver operating characteristic (ROC) curves assessed diagnostic performance, while decision curve analysis (DCA) quantified net benefits across varying threshold probabilities. Calibration curves were employed to analyze the agreement between predicted probabilities and observed outcomes. Statistical comparison of diagnostic efficacy among models was performed using DeLong’s test.

## 3. Statistical Analysis

All statistical analyses were performed using R software (v2024.04.2.0; R Foundation for Statistical Computing) and Python (v3.7; Python Software Foundation). Missing data were preprocessed via five-fold random forest imputation. Categorical variables were compared using either chi-square or Fisher’s exact tests, while continuous variables were analyzed with Mann–Whitney U or independent *t*-tests. The discriminative ability of models was assessed through ROC analysis, with performance quantified by optimal threshold metrics including AUC, sensitivity, specificity, accuracy, and F1-score. Calibration curves and DCA evaluated clinical predictive performance and utility. Statistical differences in AUC values between models were examined using DeLong’s test. The SHAP (Shapley Additive Explanations) framework provided visual interpretation of feature contributions. Statistical significance was defined as *p* < 0.05.

## 4. Results

### 4.1. Patient Characteristics

A total of 150 patients underwent laparoscopic pancreatoduodenectomy. These patients were stratified into a training cohort (n = 105, including 44 difficult cases) and an independent test cohort (n = 45, including 20 difficult cases). The overall cohort comprised 64 difficult surgeries (43%) and 86 non-difficult surgeries (57%). Demographic and intraoperative characteristics are presented in Tables S2–S4. Increased surgical difficulty demonstrated significant associations with postoperative short-term complications and perioperative outcomes, including postoperative pancreatic fistula (POPF) grade, gastrointestinal/abdominal hemorrhage, surgical site infection (SSI), Clavien-Dindo grade III complications, and ICU admission (*p* < 0.05). [12] (Supplementary Material Figure S1, Table S5).

### 4.2. Imaging Characteristics

Based on portal venous phase 3D segmentations, 1834 radiomic features were extracted from both intratumoral and peritumoral volumes of interest (VOIs). Deep learning features were captured using a ResNet18 convolutional neural network architecture incorporating pooling operations. Following LASSO feature selection, all features with non-zero coefficients were retained for regression modeling (Supplementary Material Figure S2). Features were ranked by absolute coefficient magnitude to identify the most influential predictors (Supplementary Material Figure S3).

### 4.3. Predictive Model Performance

**Figure 2:**
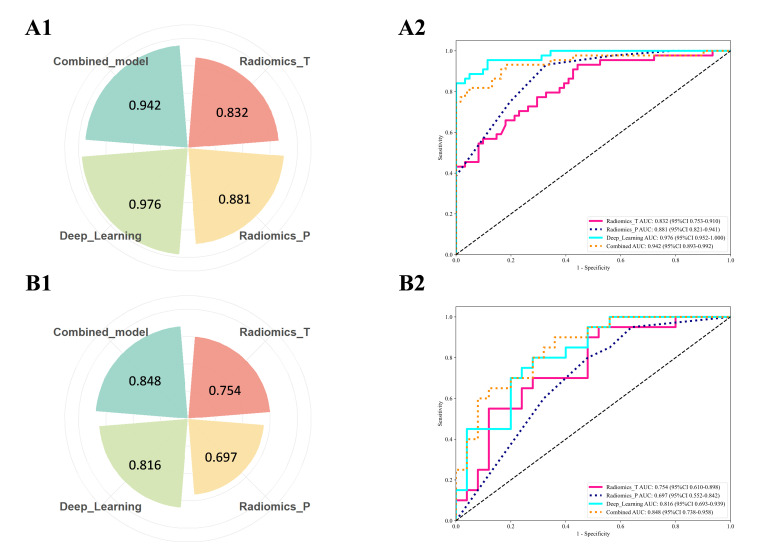
Receiver operating characteristic (ROC) curves of the four models in the training set (**A**) and validation set (**B**). Among the four models, the combined model of radiomics and deep learning had the best discriminating ability, with an area under the curve (AUC) of 0.942 in the training set and 0.848 in the validation set.

**Figure 3:**
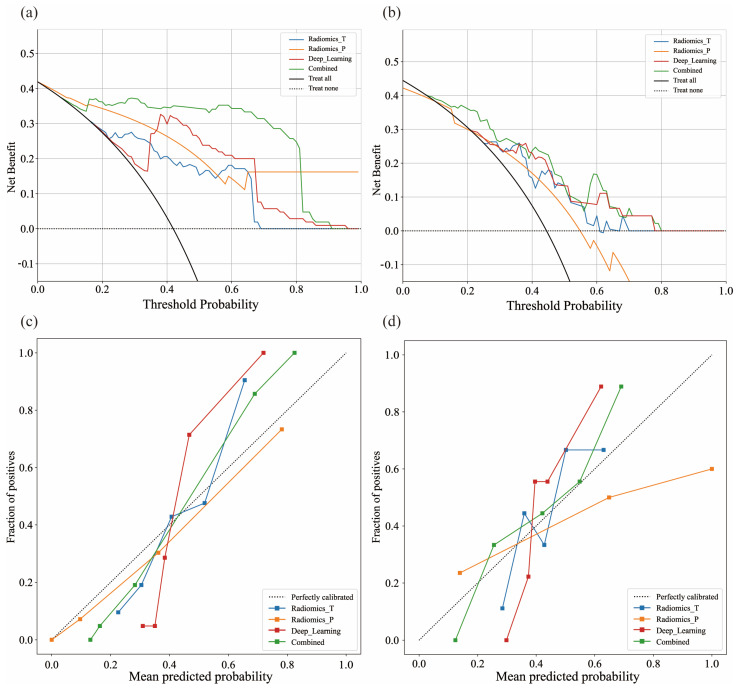
Decision curve analysis (DCA) of the combined models in the training set (**a**) and validation set (**b**). The *x*-axis indicates the high-risk threshold, and the *y*-axis indicates the clinical net benefit. Calibration curves of the combined models in the training set (**c**) and validation set (**d**). The *x*-axis represents the predicted probability calculated by the models, and the *y*-axis represents the actual probability. The black dotted line indicates the ideal evaluation by a perfect model, and the solid lines with different colors show the discrimination ability of different models. The closer a line is to the dotted line, the better the performance.

**Figure 4:**
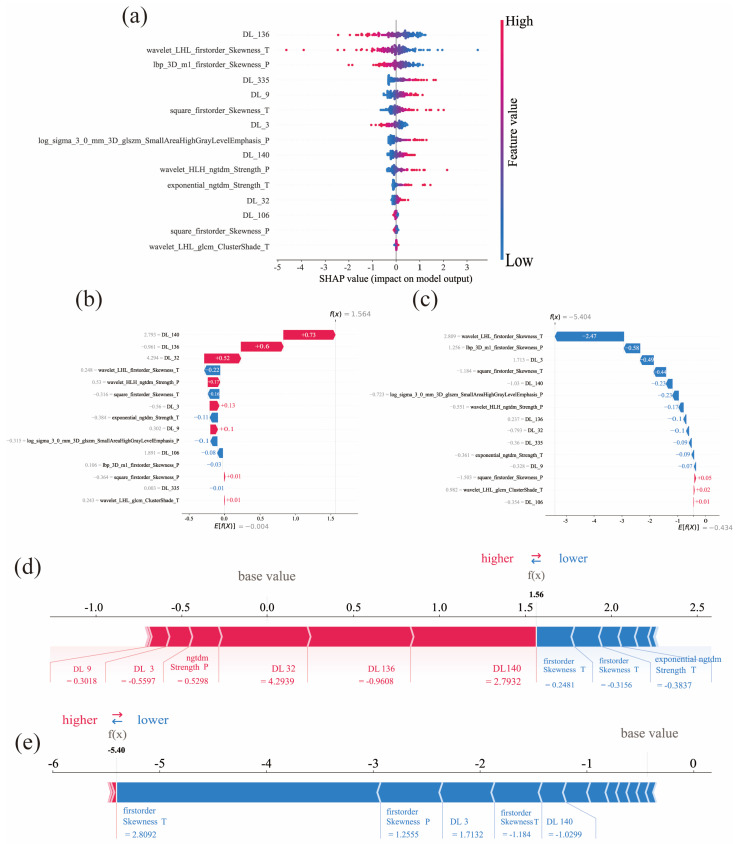
Visualization analysis; (**a**): SHAP visualization analysis shows the contribution value of each feature included in the combined model; (**b**,**c**): Waterfall plot. Each horizontal bar represents the contribution of a feature to the predicted value. The length of the bar indicates the magnitude of the feature’s impact on the predicted value, with positive contributions extending to the right and negative contributions extending to the left. From top to bottom, the contributions of each feature are successively added to the predicted value. (**d**,**e**): Force plot analysis. Red features (on the left) indicate characteristics that increase the occurrence of positive events (difficult surgery), while blue features represent characteristics that decrease the occurrence of positive events. The length of the arrows helps visualize the magnitude of the impact on the prediction.

**Figure 5:**
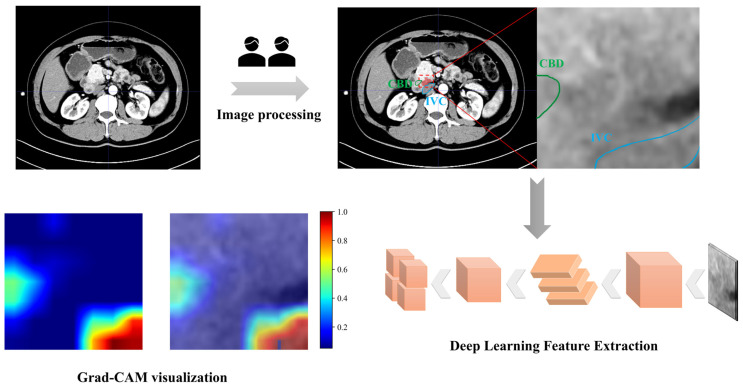
Grad-CAM visualization. CBD: Common bile duct; IVC: inferior vena cava.

Four predictive models were constructed utilizing an SVM model. No statistically significant correlation was observed between the clinical features and groups stratified by surgical difficulty in this study (*p* > 0.05). (Tables S1 and S3). To evaluate feature contributions and identify the optimal model, comparative performance assessments were conducted across training and test cohorts. The combined radiomics and deep learning model (RDLM) demonstrated superior performance, achieving a test set AUC of 0.848 (95% CI: 0.7376–0.9584), accuracy (ACC) of 0.733, and negative predictive value (NPV) of 0.860. The radiomic tumor model (RTM) exhibited high specificity (Spe: 0.880) and positive predictive value (PPV: 0.750), while the combined model showed favorable sensitivity (Sen: 0.850) and NPV (0.842) (Table 2, Figure 2). DeLong’s test results comparing model AUCs are presented in Table 3. In the training cohort, RDLM performed comparably to the deep learning model (DLM), both outperforming traditional radiomic tumor (RTM) and peritumoral (RPM) models. Within the test cohort, RDLM demonstrated the highest predictive performance among all models, though DLM did not significantly outperform conventional radiomics models. The combined model exhibited optimal calibration, with predicted probabilities closely aligning with observed outcomes across most ranges. Hosmer–Lemeshow testing confirmed no significant deviation between predicted and actual probabilities (goodness-of-fit *p* > 0.05, Table 4). Decision curve analysis further validated clinical utility (Figure 3), showing the combined model yielded the highest net benefit across most threshold probabilities, particularly above 20%. SHAP analysis elucidated the combined model’s decision patterns (Figure 4), revealing DL_136 and wavelet_LHL_firstorder_Skewness_T as the most influential features (widest SHAP value distributions), while wavelet_LHL_glcm_ClusterShade_T showed minimal impact. DL features (e.g., DL_140, DL_32) drove positive contributions to high-difficulty predictions (linking to peritumoral invasion), with radiomic features as complements. Gradient-Weighted Class Activation Mapping (Grad-CAM) visualization results demonstrated that the combined model primarily focused on peritumoral tissue and adjacent structures (e.g., the common bile duct and inferior vena cava) in the images. This aligns with the surgical principle that the core of LPD revolves around the identification and dissection of critical blood vessels and bile ducts surrounding the pancreatic head (Figure 5).

## 5. Discussion

Pancreaticoduodenectomy is one of the advanced surgeries in hepatopancreatobiliary surgery. Studies report conversion rates reaching 15–20% in high-difficulty LPD cases, associating to severe postoperative complications including pancreatic fistula and surgical site infections [37]. Therefore, accurate preoperative assessment of surgical difficulty is crucial for optimizing treatment decisions. However, the current assessment of surgical difficulty largely relies on the subjective experience of surgeons [16]. To address this limitation, assessment systems based on objective indicators have gained widespread recognition. Conversion to open surgery, estimated intraoperative blood loss (EBL), and operative time are widely recognized as classic benchmarks for assessing the difficulty of laparoscopic abdominal surgery [7,30,31,32].

Currently, studies on predicting pancreatic surgical difficulty are primarily focused on clinical and anatomical features. By analyzing 99 pancreaticoduodenectomy (PD) cases, Kosaka H et al. demonstrated that high-difficulty surgeries correlate with prolonged operative time and increased estimated blood loss (EBL). They further identified unrecognized anatomical tissue planes as an independent determinant of surgical difficulty [5]. Napoli N et al. developed the PD-ROBOSCORE scoring system, which incorporates factors including BMI, gender, tumor resectability, morphology, pancreatic duct diameter, ASA classification, and vascular variations [12]. Notably, specific local anatomical features such as mesenteric thickness and abdominal wall thickness have also been validated as effective predictors of surgical difficulty in robotic pancreaticoduodenectomy (RPD) [38].

The concept of precision surgery has driven the development of preoperative assessment systems based on multidimensional data. These systems enable quantitative stratification of surgical risks and provide a basis for individualized treatment strategies. Advances in artificial intelligence (AI) technology offer strong support for this progress. For example, AlphaFold has predicted the 3D structure of proteins from amino acid sequences, demonstrating the potential to interpret biological functions through molecular structure analysis [39]. Meanwhile, large language models have shown the ability to integrate multi-source heterogeneous data to assist in complex clinical decision-making [40]. Currently, AI algorithms are widely applied in preoperative evaluation, intraoperative decision-making, and postoperative monitoring for surgical procedures [41]. For instance, a CT-based deep learning model successfully predicted surgical complexity in patients with ventral hernias during external validation. Its performance outperformed the judgments of experienced surgeons [42]. Additionally, the DeepSurgery system—built using a 3D convolutional neural network (3D CNN) algorithm—has standardized the workflow for cataract surgery video recognition. It also enables real-time guidance and early warning for surgical performance [43]. Furthermore, a deep learning model was used to automatically segment hepatobiliary anatomical structures, establishing safety threshold criteria for assessing bile duct injury risk during laparoscopic cholecystectomy [44]. In the present study, we extracted and integrated multidimensional quantitative information from tumors and their surrounding tissues using preoperative CT images. This integration leveraged both HCR features and AI deep learning algorithms. A multi-stage feature selection strategy was adopted [25,35,45], and the retained features were capable of quantifying the heterogeneity of the peritumoral stroma [21,22], indicating the invasive growth pattern of tumors. The feature dimension utilized by the DLR model was higher than that of the HCR model. This is attributed to DLR capturing subtle microstructural abnormalities at the tumor-vascular interface [23,46]. The performance of the deep learning model (DLM) was significantly superior to that of the HCR model (AUC = 0.816), highlighting the limitations of traditional features in characterizing complex nonlinear relationships. This performance gap may stem from the insufficient generalization ability of conventional models in scenarios with limited sample sizes. From a mechanistic perspective, there is a striking contrast between the dynamic feature learning capability of DLR and the reliance of HCR on static morphological features [47].

In the field of pancreatic surgery, traditional imaging scoring systems have been widely used for predicting postoperative pancreatic fistula (POPF). Multiple studies have developed POPF risk models based on computed tomography (CT) imaging features [13]. For example, Choubey AP et al. constructed a model by integrating clinical variables and radiomic features, which exhibited excellent performance in predicting clinically relevant POPF grade B/C (training set AUC: 0.84; test set AUC: 0.78) [14]. Another study that employed deep learning score (DLS) for preoperative quantitative CT assessment also confirmed its potential in evaluating the risk of clinically relevant POPF (CR-POPF) in patients with moderate POPF risk (training set AUC: 0.85; test set AUC: 0.81) [15]. Compared with these studies, the innovation of the present study lies in the adoption of a feature fusion strategy [46], with the construction of a combined model integrating HCR and DLR features. This combined model demonstrated superior predictive performance in both the training and test sets (training set AUC = 0.942; test set AUC = 0.848). The integration may enable simultaneous quantification of key aspects of tumor biological behavior, including the tumor-vascular spatial relationship and local heterogeneity of peritumoral tissues [48].

For the clinical translation level, AI models can be applied in minimally invasive surgical training to help physicians select appropriate surgical cases based on their skill levels [49,50], avoiding undertaking surgeries with excessively high difficulty [6]. In preoperative planning, AI models can help surgeons establish more realistic expectations regarding surgical duration, potential bleeding risks, and the extent of lymph node dissection [16]. In terms of postoperative management, enhanced monitoring protocols can be initiated for high-difficulty cases, such as increasing the frequency of drainage fluid amylase monitoring and reserving channels for early intervention, which shortens the time window from the occurrence of disease changes to effective treatment [6].

SHAP visualization analysis elucidated the inherent opacity within the deep learning model’s decision-making process [51]. Peritumoral heterogeneity features (e.g., lbp_3D_m1_firstorder_Skewness_P) reflect rough-scale intensity variations, likely corresponding to stromal inflammatory infiltration and tumor invasive growth. Notably, intratumoral skewness (e.g., wavelet_LHL_firstorder_Skewness_T) shows predictive value: asymmetric intensity distribution may indicate structural disarray in high-grade malignancies [52]. As top contributors, DL features provide key information about subtle texture changes, tumor microenvironment characteristics, angiogenic hotspots and inflammatory cell infiltration [20,21,22,46]. These highly abstract nonlinear deep features explain how increased inflammatory infiltration in the surgical field raises the degree of tissue adhesion, thereby making the identification of intraoperative tissue planes more difficult and further increasing surgical complexity [9]. Grad-CAM visualization shows the regions focused on by the convolutional neural network (CNN) during the feature extraction process. The deep learning radiomics framework captures implicit expressive features from hypoenhancement patterns associated with low microvessel density and fibrous tissue hyperplasia [23]. Notably, the activation maps exhibit significant differences in signal intensity within the gastrointestinal tract, biliary structures, and vascular networks, indicating that the model has a unique ability to depict anatomical boundaries in a layered manner through deep features.

## 6. Limitations

This study also has certain limitations. Firstly, the retrospective study design may introduce selection bias. Secondly, surgical difficulty indicators are inevitably influenced by the LPD case volume of the medical center, surgeons’ operational experience, and learning curves. Kawaguchi Y et al. proposed that surgeries of different difficulty levels be performed by surgeons with varying experience levels, which partially controls the impact of surgeons’ experience on the authenticity of difficulty assessment [30]. This strategy provides a valuable reference for optimizing the difficulty classification system in the future. Additionally, limited to single-center data (n = 150), only internal validation of the model was conducted. In the future, multicenter external validation will better evaluate its generalization performance and confirm clinical feasibility. Meanwhile, a single machine learning algorithm carries overfitting risks, which need to be controlled through parameter adjustment and rigorous validation set verification. With the continuous incorporation of new data, the model is expected to be improved via continuous learning.

## 7. Conclusions

In summary, this study developed and validated an integrated CT radiomics-deep learning model for preoperative prediction of LPD surgical difficulty, which exhibits high sensitivity in identifying high-difficulty cases. Its key advantages include non-invasiveness, robust calibration, and considerable clinical net benefit, holding significant value for guiding surgeons in surgical decision-making.

## Figures and Tables

**Table 1:** Definition of the difficulty surgical score for laparoscopic pancreaticoduodenectomy.

The Difficulty Score System for Laparoscopic Pancreaticoduodenectomy					
Duration of surgery	>492 min (P75)	1	Total score	0	Non-difficult group
Conversion to open procedure	True	1			
				≠0	Difficult group
Intraoperative blood loss	>300 mL (P75)	1			

P75: The 75th percentile interquartile range of the study cohort.

**Table 2:** Performance of four models in the training set and validation set.

Cohort	Model	AUC	AUC 95%CI	Acc	Acc 95%CI	Sen	Spe	PPV	NPV	Precision	Recall	F1	Cutoff
Train	Radiomics_T	0.832	0.7531–0.9101	0.705	0.6078–0.7898	0.909	0.557	0.597	0.895	0.597	0.909	0.721	0.339
Train	Radiomics_P	0.881	0.8205–0.9406	0.781	0.6897–0.8558	0.750	0.803	0.733	0.817	0.733	0.750	0.742	0.581
Train	Deep_Learning	0.976	0.9518–0.9998	0.924	0.8554–0.9665	0.818	1.000	1.000	0.884	1.000	0.818	0.900	0.594
Train	Combined	0.942	0.8926–0.9919	0.886	0.8089–0.9395	0.795	0.951	0.921	0.866	0.921	0.795	0.854	0.412
Test	Radiomics_T	0.754	0.6101–0.8979	0.689	0.5335–0.8183	0.450	0.880	0.750	0.667	0.750	0.450	0.562	0.500
Test	Radiomics_P	0.697	0.5518–0.8422	0.644	0.4878–0.7813	0.600	0.680	0.600	0.680	0.600	0.600	0.600	0.649
Test	Deep_Learning	0.816	0.6931–0.9389	0.733	0.5806–0.8540	0.750	0.720	0.682	0.783	0.682	0.750	0.714	0.412
Test	Combined	0.848	0.7376–0.9584	0.733	0.5806–0.8540	0.850	0.640	0.654	0.842	0.654	0.850	0.739	0.387

**Table 3:** DeLong test showed that the combined model had better performance than other models in the validation set.

Cohort	Combined vs. DL	Combined vs. Radiomics_P	Combined vs. Radiomics_T	Radiomics_P vs. Radiomics_T	DL vs. Radiomics_T	DL vs. Radiomics_P
Train	0.167	0.034	0.003	0.309	<0.001	0.005
Test	0.025	0.047	0.013	0.570	0.495	0.182

**Table 4:** Hosmer–Lemeshow test demonstrated that the combined model exhibited a satisfactory fit in both the training and validation sets.

Cohort	Radiomics_T	Radiomics_P	Deep_Learning	Combined
Train	0.389	0.199	0.164	0.314
Test	0.396	0.012	0.356	0.307

## Data Availability

The original contributions presented in the study are included in the article material, and further inquiries can be directed to the corresponding author. Due to ethical restrictions and patient confidentiality regulations, the CT imaging data are not publicly available.

## Supplementary Materials

The following supporting information can be downloaded at: https://www.mdpi.com/article/10.3390/cancers18010029/s1, Figure S1: Double-layered pie chart of perioperative outcomes between the difficult and non-difficult groups in laparoscopic pancreaticoduodenectomy (LPD). Figure S2: Process of radiological feature selection. Figure S3: Distribution of feature weights in different models. Table S1: Univariate Logistic Regression Analysis of Clinical Characteristics. Table S2: Baseline Characteristics of the Training and Test Cohorts. Table S3: Clinical features in the difficult and non-difficult groups. Table S4: Comparison of surgical information among different surgical difficulty groups in LPD. Table S5: Comparison of perioperative outcomes between the difficult and non-difficult groups in LPD.

## Abbreviations

LPD	Laparoscopic pancreaticoduodenectomy
HCR	Hand-crafted radiomics
DLR	Deep learning-derived radiomics
GTV	Gross tumor volume
GPTV	Gross peri-tumor volume
SSI	Surgical site infections
POPF	Postoperative pancreatic fistula
ROI	Regions of interest
GLCM	Gray-Level Co-occurrence Matrix
GLRLM	Gray-Level Run Length Matrix
GLDM	Gray-Level Dependence Matrix
GLSZM	Gray-Level Size Zone Matrix
NGTDM	Neighborhood Gray-Tone Difference Matrix
SHAP	Shapley Additive Explanations
RDLM	Radiomics and deep learning model
DLM	Deep learning model
RTM	radiomic tumor model
RPM	peritumoral model
PCA	Principal Component Analysis
