# Supplementary material for: Development and Validation of a CT Radiomics-Deep Learning Model for Predicting Surgical Difficulty in Pancreatic and Periampullary Tumors
Source: Cancers (Basel). 2025 Dec 21;18(1):29. doi: 10.3390/cancers18010029 (PMC12784925; doi:10.3390/cancers18010029)
Supplement: Supplementary file 1 [file cancers-18-00029-s001.zip › cancers-4025994-supplementary.pdf]

Supplementary material_1. Double-layered pie chart of perioperative outcomes between the difficult and non-difficult groups in laparoscopic pancreaticoduodenectomy (LPD)

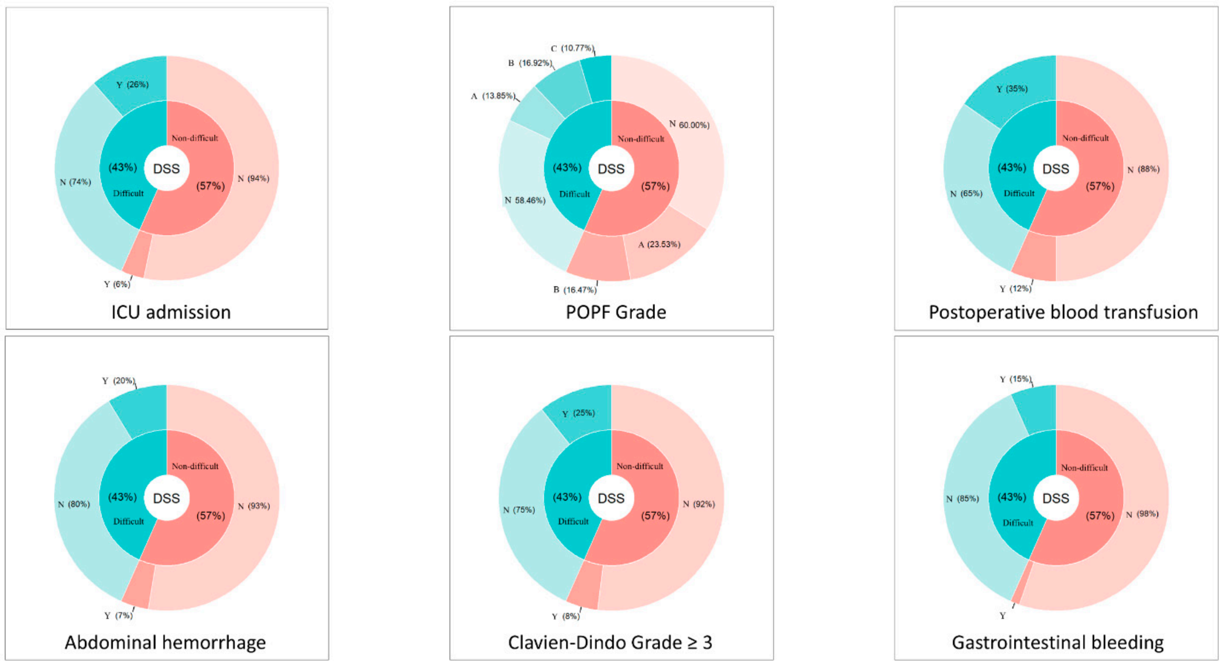

Supplementary material_2. Process of radiological feature selection

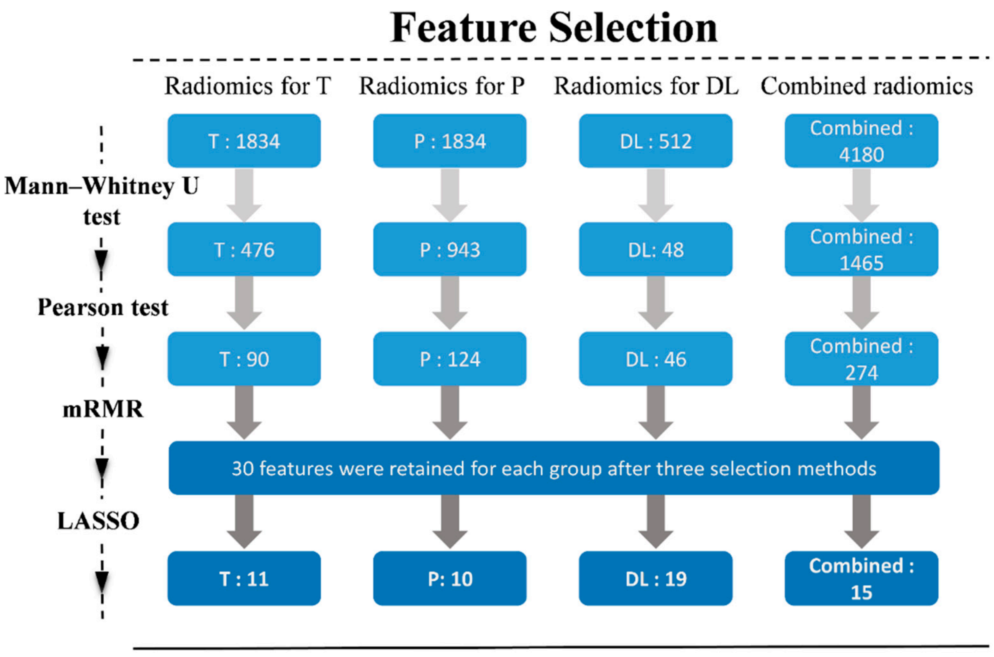

Supplementary material_3. Distribution of feature weights in different models

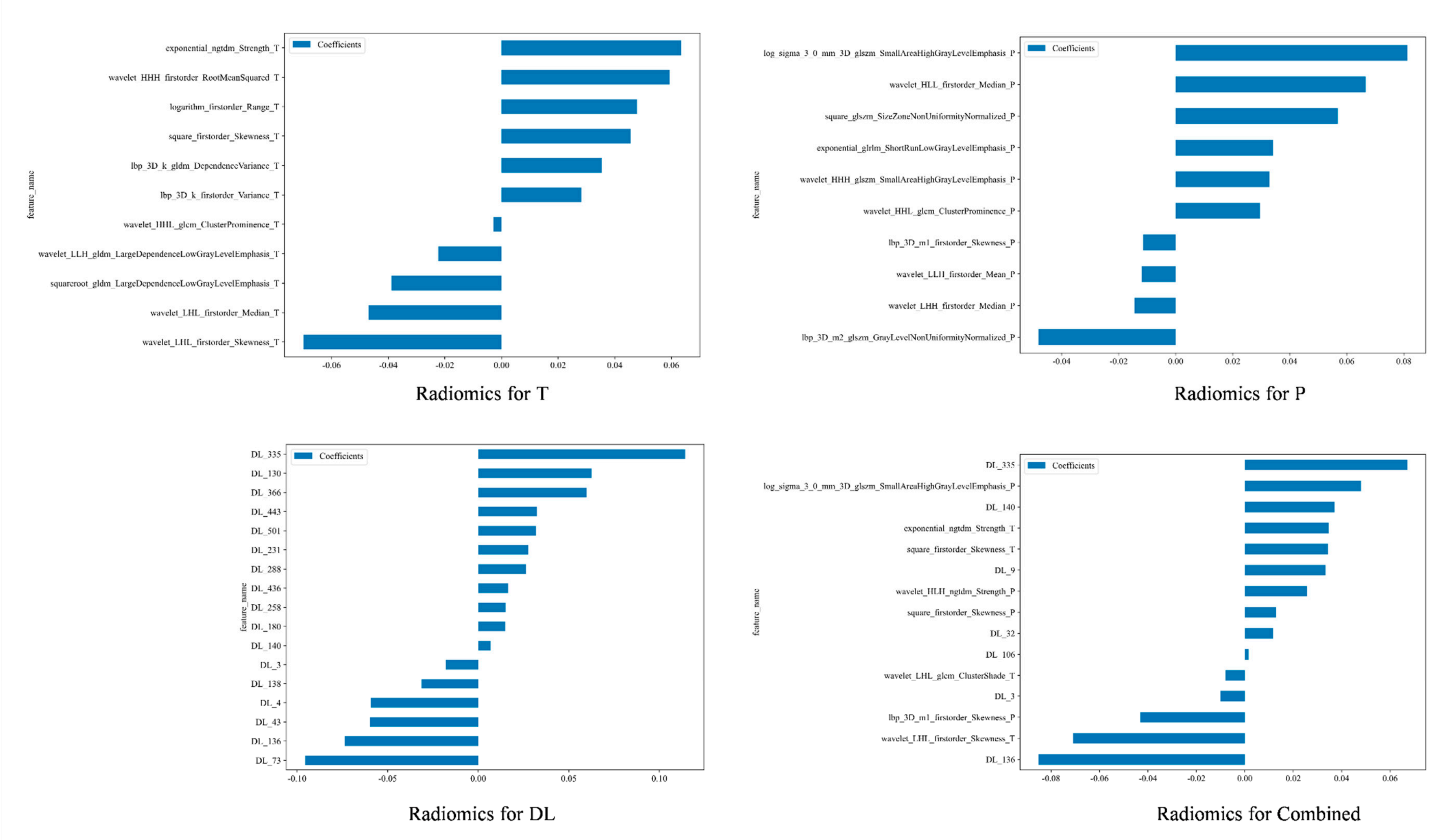

Table_S1. Univariate Logistic Regression Analysis of Clinical Characteristics

Feature	OR	OR lower 95%CI	OR upper 95%CI	p_value
**CA199**	0.945	0.784	1.139	0.617
**Diagnose**	0.969	0.903	1.040	0.456
**NLR**	0.990	0.975	1.006	0.294
**BMI**	0.994	0.969	1.019	0.678
**Age**	0.996	0.988	1.004	0.462
**PLR**	1.000	0.999	1.000	0.480
**SII**	1.000	1.000	1.000	0.496
**PAR**	1.002	0.967	1.039	0.920
**ASA**	1.009	0.865	1.176	0.927
**CEA**	1.140	0.925	1.404	0.300
**Gender**	1.194	1.013	1.408	0.077

Inflammatory Markers: (1) NLR = N/L; (2) PLR = PLT/L; (3) SII (Systemic Immune-Inflammation Index) = N×PLT/L; (4) PAR = PLT/Alb (PLT: Platelet, 10^9/L; N: Neutrophil, 10^9/L; Alb: Albumin, g/L; L: Lymphocyte, 10^9/L)

Table_S2. Baseline Characteristics of the Training and Test Cohorts

Variables	Total (n = 150)	Train (n = 105)	Validation (n = 45)	p
Group, n (%)				0.914
Non-Difficult	86 (57)	61 (58)	25 (56)	
Difficult	64 (43)	44 (42)	20 (44)	
ASA, n (%)				0.099
I	1 (1)	1 (1)	0 (0)	
II	57 (38)	34 (32)	23 (51)	
III	89 (59)	68 (65)	21 (47)	
IV	3 (2)	2 (2)	1 (2)	
Age, Mean ± SD	61.4 ± 9.48	61.66 ± 10.03	60.8 ± 8.12	0.583
Sex, n (%)				0.511
f	59 (39)	39 (37)	20 (44)	
m	91 (61)	66 (63)	25 (56)	
BMI, Mean ± SD	22.59 ± 3.24	22.77 ± 3.15	22.19 ± 3.45	0.333
Abdominal surgery history, n (%)				0.504
0	120 (80)	82 (78)	38 (84)	
1	30 (20)	23 (22)	7 (16)	
Positive surgical margin, n (%)				1
0	149 (99)	104 (99)	45 (100)	
1	1 (1)	1 (1)	0 (0)	
AFP, Median (Q1,Q3)	2.9 (2.1, 3.98)	2.9 (2.1, 3.9)	3.1 (2.3, 4.4)	0.449
CEA, Median (Q1,Q3)	3.4 (2.1, 4.88)	3.3 (1.9, 4.6)	3.4 (2.6, 5.6)	0.184
CA199, Median (Q1,Q3)	98.2 (31, 394.55)	105.6 (31, 391.7)	90.9 (34.8, 430.3)	0.959
Diagnose, n (%)				0.877
Pancreatic tumor	91 (61)	65 (62)	26 (58)	
Distal cholangiocarcinoma	25 (17)	16 (15)	9 (20)	
Duodenal tumor	9 (6)	6 (6)	3 (7)	
Ampullary tumor	25 (17)	18 (17)	7 (16)	

ASA: American Society of Anesthesiologists; f: female; m: male

Table_S3. Clinical features in the difficult and non-difficult groups

Variables	Total (n=150)	Non-Difficult (n=85)	Difficult (n=65)	p
ASA, n (%)				0.303
I	1 (1)	1 (1)	0 (0)	
II	58 (39)	35 (41)	23 (35)	
III	89 (59)	49 (58)	40 (62)	
IV	2 (1)	0 (0)	2 (3)	
Age, Mean ± SD	61.4 ± 9.48	61.72 ± 10.07	60.97 ± 8.71	0.623

Sex, n (%)				0.041
f	59 (39)	40 (47)	19 (29)	
m	91 (61)	45 (53)	46 (71)	
High, Median (Q1,Q3)	162 (156, 168)	161 (155, 168)	164 (158, 168)	0.111
Weight, Mean ± SD	59.43 ± 10.19	58.49 ± 10.36	60.66 ± 9.89	0.196
BMI, Mean ± SD	22.59 ± 3.24	22.51 ± 3.44	22.7 ± 2.99	0.722
Abdominal surgery history, n (%)				0.303
No	120 (80)	65 (76)	55 (85)	
Yes	30 (20)	20 (24)	10 (15)	
Positive surgical margin, n (%)				0.433
0	149 (99)	85 (100)	64 (98)	
1	1 (1)	0 (0)	1 (2)	
AFP, Median (Q1,Q3)	2.9 (2.1, 3.98)	3.1 (2.2, 4.4)	2.7 (2.1, 3.9)	0.287
CEA, Median (Q1,Q3)	3.25 (2.1, 4.88)	2.9 (2.1, 4.4)	3.5 (2.5, 5)	0.146
CA199, Median (Q1,Q3)	105.75 (31.62, 418.08)	92.7 (31.4, 301.1)	147.3 (32.5, 583.7)	0.404
Diagnose, n (%)				0.992
Pancreatic tumor	91 (61)	51 (60)	40 (62)	
Distal cholangiocarcinoma	25 (17)	14 (16)	11 (17)	
Duodenal tumor	9 (6)	5 (6)	4 (6)	
Ampullary tumor	25 (17)	15 (18)	10 (15)	

ASA: American Society of Anesthesiologists; f: female; m: male

Table_S4. Comparison of surgical information among different surgical difficulty groups in LPD

	Total (n = 150)	Non-Difficult (n = 85)	Difficult (n = 65)
Surgery, n (%)			
LOPD	19 (13)	0 (0)	19 (29)
LPD	117 (78)	76 (89)	41 (63)
RLPD	14 (9)	9 (11)	5 (8)
Surgtime(min), Median (Q1,Q3)	430 (367, 490)	380 (350, 425)	490 (445, 545)
Surgbleed(ml), Median (Q1,Q3)	200 (200, 300)	200 (150, 200)	300 (200, 500)

LOPD: Laparoscopic converted pancreaticoduodenectomy; RLPD: Robotic laparoscopic pancreaticoduodenectomy; Surgtime: Surgical time; Surgbleed: Surgical blood loss

Table_S5. Comparison of perioperative outcomes between the difficult and non-difficult groups in LPD

	Non-Difficult n=85	Difficult n=65	P value
POPF			0.009

No	51 (60.0%)	38 (58.5%)	
1	20 (23.5%)	9 (13.8%)	
2	14 (16.5%)	11 (16.9%)	
3	0 (0.00%)	7 (10.8%)	
Gastro-bleed			0.009
No	83 (97.6%)	55 (84.6%)	
Yes	2 (2.35%)	10 (15.4%)	
Abdominal bleed			0.035
No	79 (92.9%)	52 (80.0%)	
Yes	6 (7.06%)	13 (20.0%)	
Post-sugtrans			0.001
No	75 (88.2%)	42 (64.6%)	
Yes	10 (11.8%)	23 (35.4%)	
SSI			0.003
No	78 (91.8%)	47 (72.3%)	
Yes	7 (8.24%)	18 (27.7%)	
Reoperation			0.099
No	80 (94.1%)	55 (84.6%)	
Yes	5 (5.88%)	10 (15.4%)	
Complication grade >3			0.011
No	78 (91.8%)	49 (75.4%)	
Yes	7 (8.24%)	16 (24.6%)	
ICU			0.001
No	80 (94.1%)	48 (73.8%)	
Yes	5 (5.88%)	17 (26.2%)	

	Total (n = 150)	Non-Difficult (n = 85)	Difficult (n = 65)	p
Length of stay Median (Q1, Q3)	26 (20, 29)	22 (19, 27)	28 (25, 33)	< 0.001
postsugday, Median (Q1, Q3)	16 (13.25, 20)	15 (13, 17)	19 (16, 25)	< 0.001

POPF: postoperative pancreatic fistula; Gastro-bleed: Gastrointestinal bleed; Post-sugtrans: postoperative transfusion; SSI: surgical site infection; Postsugday: postoperative hospital day.
